# The Histopathology of Labial Salivary Glands in Primary Sjögren's Syndrome: Focusing on Follicular Helper T Cells in the Inflammatory Infiltrates

**DOI:** 10.1155/2014/631787

**Published:** 2014-08-07

**Authors:** Krisztina Szabo, Gabor Papp, Balazs Dezso, Margit Zeher

**Affiliations:** ^1^Division of Clinical Immunology, Medical Center, University of Debrecen, Moricz Zs. Street 22, Debrecen 4032, Hungary; ^2^Department of Pathology, Medical Center, University of Debrecen, Nagyerdei Boulevard 98, Debrecen 4032, Hungary

## Abstract

Recently, we revealed the importance of follicular helper T cells (T_FH_) in the pathogenesis of primary Sjögren's syndrome (pSS). In the present study, we focused on the site of the inflammation and determined the composition of lymphocyte infiltration in labial salivary gland (LSG) biopsies with special emphasis on T_FH_ and germinal center B cells. We selected tissue blocks obtained from ten patients at the time of disease onset. Detection of cell specific markers was performed with immunohistochemical and immunofluorescence stainings. We evaluated patients' clinical and laboratory features retrospectively and assessed the relation between disease course and early histopathological findings. LSG biopsies were graded based on the extension and arrangement level of periductal inflammatory cell infiltrates. T_FH_ cell markers (CD84, PD-1, and Bcl-6) occurred predominantly in more organized structures with higher focus scores. The coexpression of CD3 and Bcl-6 markers clearly identified T_FH_ cells close to Bcl-6^+^ B cells with the typical formation of germinal centers. Systemic features were developed later in the disease course only in patients with highly structured infiltrates and the presence of T_FH_ cells. Our observations suggest that the presence of T_FH_ cells in LSGs at the disease onset may predict a more pronounced clinical course of pSS.

## 1. Introduction

Primary Sjögren's syndrome (pSS), also known as autoimmune epithelitis, is a common chronic autoimmune disease characterised by the inflammation of exocrine glands and the clinical signs of xerostomia and keratoconjunctivitis sicca. A combination of environmental, genetic, and possibly hormonal factors leads to the dysregulation of the glandular epithelium, mononuclear cell infiltration, and abnormal lymphocyte activation and proliferation [1, 2]. Aberrant humoral autoimmune responses, B cell hyperactivity, and autoantibody production are the hallmarks of pSS [3–5].

Follicular helper T (T_FH_) cells are specialized subsets of effector T cells that provide essential help to antigen specific B cells in the secondary lymphoid organs. T_FH_ cells are originated from naive CD4^+^ T cells which are activated by dendritic cells (DCs) in the interfollicular or T cell zones [6, 7]. As a result of the initial interaction with DCs, primed CD4^+^ T cells migrate to the border of T and B cell areas and become pre-T_FH_ cells. This follicular homing process is directed by Bcl-6, which coordinates the downregulation of T cell zone homing C-C chemokine receptor type 7 (CCR7) in parallel with the upregulation of B cell region homing C-X-C chemokine receptor 5 (CXCR5) [8–12]. At the border of T and B cell areas, the interaction between pre-T_FH_ cells and activated B cells is crucial for both the generation of antibody-producing extrafollicular plasmablasts and the formation of germinal centers (GCs). In order to enter GCs, pre-T_FH_ cells require mutual signals from activated B cells via CD28-CD86, ICOS-ICOSL, CD40L-CD40, programmed cell death protein-1 (PD-1)-PD-1L, and OX40-OX40L as well as signaling lymphocytic activation molecule (SLAM) family members [13–17]. In GCs, the interplay between T_FH_ and GC B cells is bidirectional; survival signals, completed with interleukin (IL)-21, are important not only for B cell survival, proliferation, and differentiation but also for the maturation of T_FH_ cells [18, 19]. The upregulation of Bcl-6 in activated GC B cells supports their survival and extremely high proliferation rate and additionally leads to the activation-induced cytidine deaminase (AID) mediated somatic hypermutation (SHM) in the dark zone of GCs [20]. Through the subsequent stimulation of CD40 by T_FH_ cells, centroblasts differentiate into centrocytes and move to the light zone [21]. Follicular DCs (FDCs) and T_FH_ cells promote the positive selection and possible immunoglobulin class-switch recombination (CSR) of several centrocytes resulting in their differentiation into high-affinity memory B cells and long-lived plasma cells [22].

Recent studies highlighted the role of T_FH_ cells in the pathogenesis of different autoimmune conditions, including systemic lupus erythematosus, Sjögren's syndrome, rheumatoid arthritis, juvenile dermatomyositis, myasthenia gravis, and autoimmune thyroid disorders [23–28]. In our previous work, we demonstrated elevated circulating T_FH_ cell percentages in pSS and revealed the importance of this cell type in the pathogenesis of the disease [29]. Despite the increased research activity in this field, the molecular mechanisms and the function of T_FH_ cells are still not known in detail. In order to extend the current knowledge, in the present study we focused on the site of the inflammation and assessed the composition of lymphocyte infiltration in labial salivary gland (LSG) biopsies with a special emphasis on the presence and potential importance of T_FH_ cells at the time of disease onset.

## 2. Materials and Methods

### 2.1. Patients

In the present study, we enrolled ten female patients (mean age ± SD: 57.2 ± 11.4) with pSS, who had been diagnosed and followed up regularly in the outpatient clinic for systemic autoimmune diseases at the Division of Clinical Immunology, University of Debrecen. The diagnosis of pSS was established according to the European-American Consensus Group criteria (AECG) [30]. The diagnosis of the patients was confirmed with positive LSG biopsy at the disease onset. None of them had evidence of malignant lymphoma or showed EGMs at the time of the pathological sampling. Three individuals, who complained of only mild sicca symptoms without fulfilling diagnostic criteria, served as controls for the histological evaluation. All patients underwent extensive clinical and serological examinations during the follow-up. Data were obtained retrospectively from their records which contained detailed information on symptoms, physical conditions, and laboratory and other findings. Anti-SSA/Ro and anti-SSB/La autoantibodies were determined by ELISA technique with AUTOSTAT II kits (Hycor Biomedical, Indianapolis, IN, USA) according to the manufacturer's instructions. The titers of serum immunoglobulin (Ig)G, IgA, and IgM were analyzed by turbidimetric immunoassay (DIALAB GmbH, Neudorf, Austria). At the end of the follow-up, circulating T_FH_-like cells were determined by CD4, CXCR5, ICOS, and PD-1 cell surface molecules and were assessed using BD FACS Calibur flow cytometer (Becton Dickinson, Franklin Lakes, NJ), as described previously [29].

Informed written consent was given by patients for their clinical records and archived biopsy samples to be used in this investigation. The study has been approved by the local Ethics Committees (Debrecen, Hungary) in 2012 (Reference number: IX-R-052/00016-22/2012.). All experiments carried out were in compliance with the Declaration of Helsinki.

### 2.2. LSG Samples and Conventional Histological Analysis

Formalin-fixed, paraffin-embedded (FFPE) tissue blocks were obtained from the archives of the Department of Pathology, University of Debrecen, which had been previously collected for routine diagnostic purposes in years 2001–2010. Four-*μ*m thick serial sections of LSG tissue specimens were prepared and stained with haematoxylin-eosin (HE) for conventional histopathological examination. The determination of focus score (FS) was based on the degree of lymphocytic infiltration in the whole biopsy. The focus score was defined as the group of inflammatory cell aggregates containing at least 50 mononuclear cells per 4 mm^2^ of tissue area. It was classified as FS = 0: no lymphocytic infiltration; FS = 1: less than 1 lymphocytic focus per 4 mm^2^; FS = 2: less than 2 lymphocytic foci per 4 mm^2^; FS = 3: two or more lymphocytic foci per 4 mm^2^ [31].

### 2.3. Immunohistochemistry

Immunohistochemical (IHC) staining was performed on serial sections of tissue blocks using standard methods [32]. Briefly, 4 *μ*m thick FFPE sections were deparaffinized, rehydrated on descending ethanol dilutions, and treated with 3% H_2_O_2_ to block endogenous peroxidase. For antigen retrieval, sections were heated in boiling citrate buffer (pH 6.0) or Tris/EDTA buffer (pH 9.0) for 3 min using a pressure cooker. After cooling, the slides were incubated with primary antibodies for 1 hour at room temperature. The following monoclonal antibodies were (mAb) used in the procedure: CD4, clone 1F6 mouse mAb (Novocastra, Leica Biosystems, Nussloch, Germany); CD5, clone 4C7 mouse mAb (Novocastra); CD20, clone L26 mouse mAb (Dako, Glostrup, Denmark); CD84, clone EPR8325 rabbit mAb (Abcam, Cambridge, UK); CD138, clone MI15 mouse mAb (Dako); PD-1, clone NAT mouse mAb (Abcam); Bcl-6, clone PG-B6p mouse mAb (Dako). Biotin-free Envision/HRP (Dako) system as secondary Ab with very intense purple (VIP) peroxidase substrate (Vector Laboratories, Peterborough, UK) was used for detection. The sections were then counterstained with methyl green (Vector Laboratories). The stained tissue samples were digitalized using Pannoramic MIDI digital slide-scanner (3D-Histech Co., Budapest, Hungary) utilizing Zeiss Plan-Apochromat objective (magnification: 20x/0.8 numerical aperture) and Hitachi (HV-F22CL) 3CCD progressive scan color camera (resolution: 0.2325 *μ*m/pixel). Image analysis was performed by the HistoQuant application of Pannoramic Viewer software 1.15.2. (3D-Histech). If applicable, at least 4 (ranging from 2 to 6) lymphocytic foci were selected randomly in each specimen per patient for analytic measurements and photodocumentation. Field area (FA; overall field area in mm^2^) and mask area (MA; overall mask area in mm^2^) were computed by the software. The FA represents the whole area of the marked infiltrates, while the MA indicates the cell-specific marker positive area. The relative MA (rMA) values were calculated as MA/FA multiplied by 100.

### 2.4. The Characterization of Periductal Cellular Infiltrates

The organizational levels of each lymphocytic infiltrate were graded by IHC staining of serial sections using CD4 and CD20 cell markers. A small number of distributed perivascular and intraepithelial lymphocytes were graded as (1); mild lymphocytic aggregates without clear organization of separate T and B cell zones were defined as grade (2); more organized lymphoid follicles were classified as grade (3); aggregates with the highest level of arrangement, which displayed distinct T and B cell regions, were graded as (4). The latter organization was also characterised by an extensive FDC network detected with CD21 marker in the center of the lymphoid aggregates, whose pattern corresponded to ectopic GC structures.

### 2.5. Immunofluorescence Staining

Double immunofluorescence (IF) staining for Bcl-6 in combination with CD3 (clone LN10, mmAb, Novocastra) or CD20 was carried out with sequential immunostaining, as described previously [32]. Sections were prepared and antigens were unmasked as detailed above. After 1-hour treatment with anti-Bcl-6 primary Ab, the slides were incubated using anti-mouse IgG(Fab)_2_ as secondary Ab coupled to polymer-HRP (Dako), followed by a tetramethylrhodamine- (TMR-) conjugated tyramide reagent of the fluorescent amplification kit (TSR-TMR System, Perkin Elmer Life Science, Boston, MA, USA) to visualize the red nuclear fluorescence. The second layer of the double IF staining was applied with anti-CD3 or anti-CD20 primary Abs plus biotinylated anti-mouse secondary IgG F(ab')_2_ followed by streptavidin-fluorescein isothiocyanate (FITC). Nuclear counterstaining was made with DAPI (blue fluorescence, Vector Laboratories). Images were obtained using a Zeiss AXIO Imager Z2 microscope (Carl Zeiss Microscopy GmbH, Jena, Germany) equipped with the following objectives: 10x/0.3 NA; 20x/0.5 NA. For transferring and editing images, Isis software (MetaSystems Group Inc., Newton, MA, USA) and Adobe Photoshop CS5 version 12.0 were used.

## 3. Results

### 3.1. Systemic Characteristics of the Study Population during the Course of the Disease

The mean age at the time of the diagnosis was 50.80 ± 10.34 and the total duration of follow-up was 7.40 ± 3.10 years. We evaluated their clinical and serological features retrospectively and assessed the relation between laboratory results, disease course, and the early histopathological findings. Data of patients enrolled in the study are detailed in Table 1. We divided patients into two groups based on their FSs. Three patients formed the group of pSS with FS = 2 and 7 patients belonged to the group of pSS with FS = 3. None of the patients had FS < 2. Peripheral T_FH_-like cell percentages were tendentiously elevated at the end of follow-up in patients with higher FS at disease onset (mean percentages of pSS with FS = 3 versus controls: 0.86% ± 0.38 versus 0.32% ± 0.12, and pSS with FS = 3 versus pSS with FS = 2: 0.86% ± 0.38 versus 0.33% ± 0.08, resp.). Importantly, systemic features such as polyarthritis (*n* = 3), Raynaud's syndrome (*n* = 2), lymphadenopathy (*n* = 1) and fibrosis pulmonum (*n* = 1), and associated diseases including primary biliary cirrhosis (*n* = 1) or primary sclerosing cholangitis (*n* = 1) developed later in the disease course only in patients with FS = 3.

### 3.2. Histological Classification of LSG Biopsies according to Focus Scoring and Grading of the Inflammatory Infiltrates

**Figure 1:**
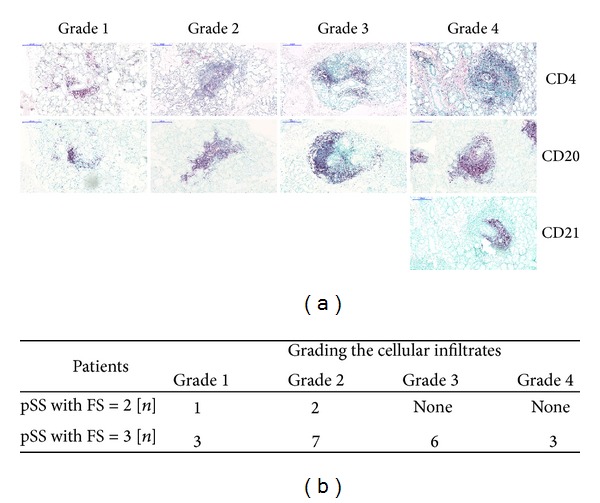
The classification of periductal inflammatory infiltrates with different levels of organization. (a) Immunohistochemical stainings of FFPE sections from representative examples of LSG sections. For rating the periductal lymphocytic infiltrates, paraffin specimens were stained for CD4, CD20, and in some cases CD21. We distinguished four different grades. Grade 1 displayed scattered T and B cells around the ducts. Grades 2 and 3 showed mild and more organized lymphocytic aggregates. Grade 4 indicated a highly organized structure with extensive FDC network in the center. The magnification of digitalized slides is 10x. Scale bar 200 *μ*m. (b) Distribution of different organizational levels in patients with pSS. *n*: number of biopsy specimens contained at the stage of organization.

When studying the morphology of LSG specimens in patients with pSS, we identified different organizational levels of inflammatory mononuclear cell infiltrates. The whole LSG specimen was characterized based on the FS, while the extension and the structural arrangement level of each periductal cellular infiltrate were graded within the biopsy section. As displayed in Figure 1(a), four distinct categories could be identified. In our study, the biopsy samples with FS = 2 consisted of lymphocytic aggregates only graded as 1 or 2. More organized follicles as grade 3 or 4 were exclusively found in pSS with FS = 3. Grade 4 lymphocytic foci exhibited features of GCs within secondary lymphoid organs.  Figure 1(b) presents the distributions of the four distinguished levels of cellular arrangement in the two groups of patients. In many cases, biopsy specimens included cellular aggregates of different kinds of grades, particularly in higher organizational levels.

### 3.3. Immunohistochemical Characterization of Infiltrating Cells according to the Cell-Specific Markers in LSG Biopsies

**Figure 2:**
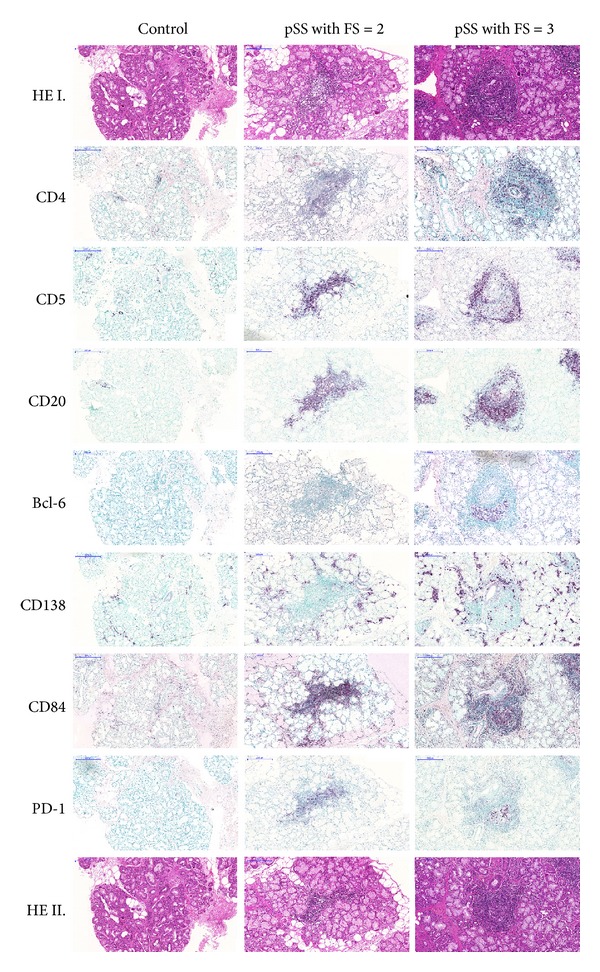
Immunohistochemical analysis of the division of T, B, and plasma cell markers with a special emphasis on T_FH_-related molecules in pSS with FS = 2 and pSS with FS = 3. Serial immunostainings of grade 2 and grade 4 aggregates show CD4^+^ T helper cells, CD5^+^ T and B1 cells, CD20^+^ B cells, and CD138^+^ plasma cells and markers which play a role in the T_FH_-B cell interaction, namely, CD84, PD-1, and Bcl-6. The magnification of digitalized slides is 10x. Scale bar 200 *μ*m.

In the biopsy samples of patients with FS = 2, we observed only a mild or moderate degree of periductal lymphocytic infiltration. In pSS with FS = 3, the infiltrations were extensive and penetrated the ductal epithelia with occasional destruction of the acini. Furthermore, three patients from pSS group with FS = 3 also had ectopic GC formation in LSG samples. Serial immunostainings for the incidence and densities of inflammatory cell-specific markers within the infiltrates of the subgroups are demonstrated in Figure 2. As shown, cell surface markers including CD4, CD5, CD20, CD138, CD84, and PD-1 were found in both groups, albeit in different arrangements and densities.

In the aggregates of pSS group with FS = 2, mainly the T helper cell marker CD4, the pan-T cell and B1 cell marker CD5, and the pan-B cell marker CD20 were detected, while the T_FH_-related markers CD84 and PD-1 were less evident. Cells characterized by the above-mentioned molecules showed scattered distribution within the infiltrates. The CD138^+^ plasma cells were dispersed throughout the whole LSG samples and found mostly outside the aggregates.

In pSS group with FS = 3, the distribution of specific cell markers showed a different pattern along with more organized structures. CD4^+^ T cells were predominantly localized at the periphery of infiltrates. Cells penetrating the ductal epithelia were also positive for CD4. CD5 were detected mainly in the T cell regions at the periphery of mononuclear cell infiltrates and only a few cells in the B cell area were positive for it. The CD20^+^ B cells were principally situated at the central region of lymphoid follicles. Similar to pSS group with FS = 2, CD138^+^ plasma cells were also displayed as a scattered distribution outside the infiltrates; however, some of them were observed at the border of B cell zone as well. The expression of CD84 cell surface molecule was diffused throughout the inflammatory infiltrate but accumulated at the inner area. In addition, the expression of PD-1 was solely found in the location of CD20^+^ B cells. Bcl-6^+^ cells were detected exclusively in pSS with FS = 3. After analysing the pSS group with FS = 3, intragroup variances were discovered; at grade 4 organization level Bcl-6^+^ cells were clustered in the central region and expressed with higher intensity, while in grade 3 aggregates Bcl-6^+^ cells were scattered and showed much lower expression (data not shown).

**Figure 3:**
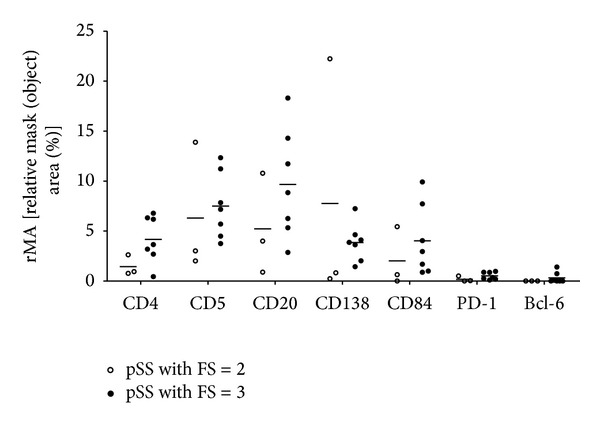
Plots indicating the distribution of T, B, and plasma cell markers with a special emphasis on T_FH_-related molecules in the inflammatory infiltrates in the two subgroups. Measurements were performed on digitalized slides with the HistoQuant module of Pannoramic Viewer software. The relative mask area is indicated in case of each marker that is presented on Figure 2. MA: overall mask area in mm^2^-summed area of each detected object in each layer; FA: overall field area in mm^2^; rMA: (MA/FA) ∗ 100, relative mask (object) area in %. Horizontal lines represent the mean value of the group.

Digitalized slide imaging allowed us to make numerical comparisons for marker expressions between the two groups. 117 slides were digitalized in total, and the studied proteins were analyzed in randomly selected lymphocytic aggregates. The average size of the aggregates in pSS with FS = 3 was larger than those in pSS with FS = 2 [0.3114 mm^2^ (0.095–0.642) versus 0.1927 mm^2^ (0.058–0.566), resp.]. As shown in Figure 3, distribution of the expression of cell-specific molecules varied according to the focus scores of biopsy samples. The expression of markers which participate in the T_FH_-B cell interaction were tendentiously higher in pSS with FS = 3.

### 3.4. Double Immunofluorescence for the Demonstration of T_FH_-Related Bcl-6 with Possible T or B Cell Coexpressions in Autoreactive Lymphocytes of LSG Biopsies

**Figure 4:**
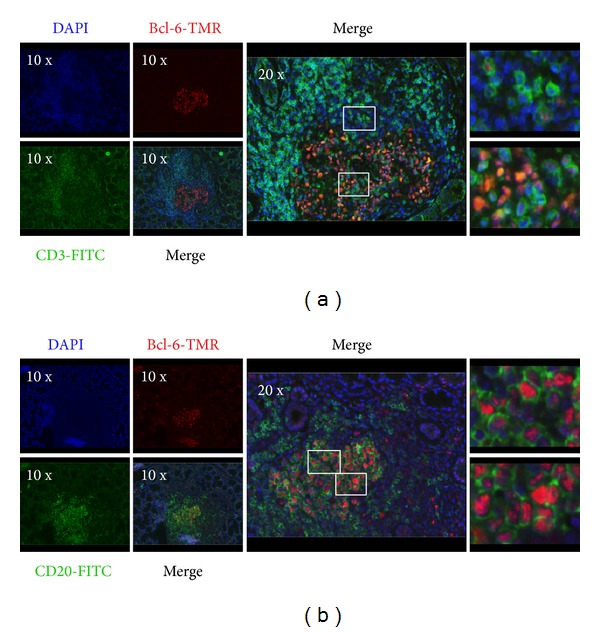
Immunofluorescence demonstration of the coexpression of CD3/CD20 and T_FH_-related molecule Bcl-6 in LSG biopsy. Double immunofluorescence stainings of LSG biopsy sections from pSS patients with Bcl-6 plus CD3 (a) and Bcl-6 plus CD20 (b). Boxed areas indicate the localization of the zoomed-in images in the right, in the same order (from top to bottom). The representative images were made from a biopsy specimen that belonged to the pSS group with FS = 3. Objectives used: 10x/0.3 NA; 20x/0.5 NA.

The last question of this study was whether Bcl-6 expression was limited to CD20^+^ B cell infiltrates of LSG or whether it could be demonstrated in CD3^+^ T cells as well. To prove that CD3^+^Bcl-6^+^ T cells were involved in the formation of GC-like structures in LSG, we stained sections by double IF for Bcl-6 and CD3 or CD20 expressions.  Figure 4(a) shows the double labeling of CD3 pan-T cell marker with the transcription factor Bcl-6 in lesional lymphocytes, indicating that a few T cells in the infiltrates were positive for Bcl-6. The coexpression of the two markers clearly identified T_FH_ cells. Bcl-6^+^ B cells with the typical formation of conventional GCs have also been detected in the central area of the lymphoid follicle demonstrated in Figure 4(b).

## 4. Discussion

Obtaining LSG biopsy is part of the routine diagnosis procedures in pSS according to the AECG, and it provides an excellent opportunity to reveal the severity of autoimmune inflammatory processes in the early stage of the disease [33, 34]. Previous studies revealed the presence of T and B cells with fewer macrophages and DCs in LSG of pSS patients [35–37]. The distribution of B cells, DCs, and FDCs correlates positively with the severity of inflammation [38]. Additionally, Foxp3^+^ cells and IL-17 and IL-21-producing cells were also detected in the infiltrates of LSG tissues [39–41]. In a recent study, Kang et al. demonstrated the coexpression of IL-21 and CXCR5 in LSG infiltrates which raised the question about the presence of T_FH_ cells [40]. Maehara et al. focused on infiltrating T lymphocyte subsets and described that the expression of T helper 2 and certain T_FH_-related molecules was associated with robust lymphocytic accumulation and ectopic GC formation [42]. Moreover, Gong et al. recently demonstrated the ability of epithelial cells to induce the differentiation of T_FH_ cells in salivary glands [43]. However, before our present investigations, T_FH_ cells were not studied in glandular lymphocytic infiltrates with different organizational levels.

In our study, we classified LSG specimens according to the severity of inflammatory cell infiltrates not only with focus scoring but also with grading of the lymphoid aggregates. To determine the FS and the grades of aggregates, we examined the entire tissue section. We observed that the biopsy samples contained different grades of mononuclear cell infiltrates, and the periductal lymphoid structures showed a higher level of organization in pSS group with FS = 3 than in pSS group with FS = 2.

**Figure 5:**
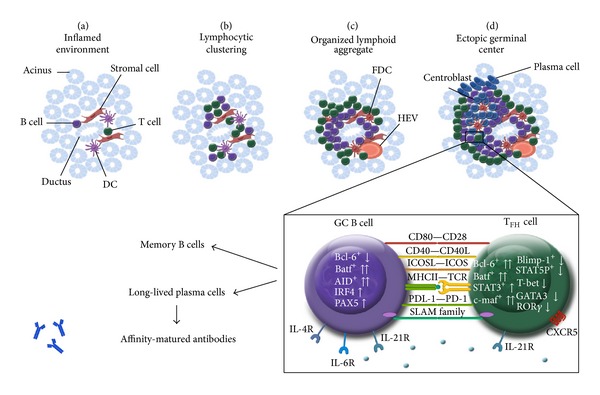
Graphical summary of ectopic lymphoid-like structure organization in exocrine tissue of patients with pSS. During the initiation phase of inflammation, activated T, B, and DC cells accumulate around ductal epithelial structures (a). Inflammatory responses, including chemokine signals by stromal cells can elicit the formation of more organized lymphoid structures (b) and (c). Aggregates with the highest organization level display separated T- and B-cell-rich areas with a central network of FDCs, which correspond to ectopic GCs (d). GC localized T_FH_ cells are characterized by CXCR5, ICOS, PD-1, CD40L, and SLAM family members as CD84, SAP, and IL-21. The interplay between T_FH_ and GC B cells is essential for the formation and maintenance of ectopic GCs, moreover for the generation of memory B cells and long-lived plasma cells. DC: dendritic cells; FDC: follicular dendritic cells; GC: germinal center; HEV: high endothelial venule; SAP: SLAM-associated protein.

Ectopic GC structures with peripheral positioned T cells, centrally localized B cell area, and a reticular pattern of FDC network were only observed in FS = 3 with grade 4 aggregates. When examining the expression of T_FH_-related molecules, such as CD84, PD-1, and Bcl-6 in the infiltrates, we found a pronounced expression in pSS with FS = 3. CD84, which is a member of SLAM family, is responsible for the maintenance of stable T-B conjugates to achieve a complete interaction and helper function by T_FH_ cells [44]. PD-1 receptor, which regulates the selection and survival of B cells in the GCs, is also an important phenotypic determinant of T_FH_ cells [45]. Marked Bcl-6 expression was detected only in grade 4 aggregates with the colocalization of B cell zone. In grade 3 infiltrates, its expression was significantly weaker. Bcl-6 expression could not be demonstrated in lower grades of aggregates at all. It is known that Bcl-6 is specially expressed by GC B cells during the centroblast phase and usually, but not consistently, in centrocytes as well [20]. According to experimental studies,* BCL6* gene defect resulted in disturbed GCs formation, with the lack of SHM and CSR, which highlights the role of Bcl-6 in GC responses [46]. Human studies also demonstrated the requirement of Bcl-6 in the establishment of GCs and found that, in contrast with aggregates, only real ectopic GCs express detectable amount of Bcl-6 [47, 48]. For that purpose, we paid a special attention to the presence and localization of T_FH_ and GC B cells in the mononuclear cell infiltration. With double IF staining, we demonstrated that, close to B cell area, a certain subset of infiltrating T cells expressed both CD3 and Bcl-6 markers, which suggests that the presence of T_FH_ cells was adjacent to GC B cells in LSG lesions. However, real GC-like structures with T_FH_ cells were merely found in those lymphoid follicles that belong to pSS group with FS = 3 and showed more severe inflammatory lesions. Our findings are in correlation with a previous study which revealed the presence of AID in lymphocytic aggregates with higher organizational level in pSS patients [49]. AID is expressed in GC B cells undergoing SHM and CSR, following the upregulation of Bcl-6. We summarized the possible role of T_FH_ cells in lymphoid aggregates in the labial salivary glands of pSS patients in a graphical figure (Figure 5).

It is important to emphasize that our investigations were performed on LSG biopsies which were collected at the time of the diagnosis, when only the initial symptoms developed in patients. The retrospective evaluation, of both laboratory and clinical data recorded during the follow-up period, revealed associations between the formation of GCs with the presence of T_FH_ cells in LSGs at disease onset and the development of EGMs and associated diseases during the disease course. Additionally, patients, who have T_FH_ cells in their salivary gland infiltrations already at the time of diagnosis, seem to also have an elevated peripheral T_FH_ cell ratio later in the disease course. It must be considered that the limitation of the present study is related to its small patient sample; thus, the correlations between the local presence of T_FH_ cells and the development of systemic clinical features cannot be justified statistically. Nevertheless, the present findings are in line with our earlier observations that the higher proportions of T_FH_ cells are associated with higher FS in glandular biopsies and the presence of extraglandular manifestations [29].

## 5. Conclusion

In the present study we demonstrated that T_FH_ cell markers, including CD84, PD-1, and Bcl-6, occur predominantly in more organized inflammatory cell infiltrates developed in LSGs with higher focus scores. Our results indicate that the presence of T_FH_ cells in LSGs at the time of disease onset may predict a more pronounced clinical course of pSS; nevertheless, this observation should be confirmed in a larger patient population as well. We expect that the further understanding of molecular and cellular regulation of T_FH_ cells will provide new potential therapeutic targets in the treatment of pSS patients with systemic manifestations.

## Figures and Tables

**Table 1:** The demographic and laboratory characteristics of patients with pSS enrolled in the study.

Patients			Laboratory findings											
No.	Age	Age at diagnosis	At diagnosis time						At present time					
			SSA/Ro	SSB/La	IgG	IgA	IgM	Focus score (FS)	SSA/Ro	SSB/La	IgG	IgA	IgM	Peripheral T_FH_-like cells (%)
			(0.00–10.00 U/mL)	(0.00–10.00 U/mL)	(7.00–16.00 g/L)	(0.70–4.00 g/L)	(0.40–2.30 g/L)		(0.00–10.00 U/mL)	(0.00–10.00 U/mL)	(7.00–16.00 g/L)	(0.70–4.00 g/L)	(0.40–2.30 g/L)	
1	67	58	176.5	117.5	40.86∗	6.34∗	2.31∗	2	120.8	51.9	6.04	2.68	0.72	0.28
2	75	66	<10	<10	9.12	1.75	1.24	2	10.4	<10	8.10	1.75	1.19	0.42
3	49	46	76.3	<10	18.09∗	1.42	2.15	2	86.6	<10	13.49	1.19	1.70	0.30
4	47	38	<10	<10	14.57	2.90	8.23∗	3	<10	<10	13.26	2.54	5.84∗	0.92
5	63	53	<10	<10	14.41	1.47	0.74	3	<10	<10	9.10	1.30	0.64	0.77
6	65	61	<10	<10	10.18	1.89	1.58	3	<10	<10	9.64	2.18	1.65	1.11
7	65	57	130.0	41.0	19.74∗	3.20	3.98∗	3	123.2	39.5	14.34	3.15	2.14	0.44
8	41	34	126.6	76.1	31.97∗	5.07∗	1.01	3	146.5	68.6	22.79∗	2.95	0.75	1.56
9	64	52	<10	<10	27.24∗	6.31∗	2.38∗	3	<10	<10	19.97∗	5.23∗	3.43∗	0.57
10	46	43	157.4	<10	26.92∗	6.34∗	2.97∗	3	157.4	<10	26.92∗	6.34∗	2.97∗	0.68

No.: number; T_FH_: follicular helper T cell; ∗higher than normal range.

## Abbreviations

AID: Activation-induced cytidine deaminase
Bcl-6: B cell lymphoma 6
CCR7: C-C chemokine receptor type 7
CSR: Class-switch recombination
CXCR5: C-X-C chemokine receptor 5
DCs: Dendritic cells
EGMs: Extraglandular manifestations
FFPE: Formalin-fixed, paraffin-embedded
FITC: Fluorescein isothiocyanate
FDCs: Follicular dendritic cells
FS: Focus score
GC: Germinal center
HE: Haematoxylin-eosin
HEV: High endothelial venule
ICOS: Inducible T cell costimulator
IL: Interleukin
IHC: Immunohistochemistry
IF: Immunofluorescence
Ig: Immunoglobulin
LSG: Labial salivary gland
PD-1: Programmed cell death protein 1
pSS: Primary Sjögren's syndrome
SAP: Signaling lymphocytic activation molecule- (SLAM-) associated protein
SHM: Somatic hypermutation
T_FH_: T follicular helper
TMR: Tetramethylrhodamine.
